# Lower-Limb Neuromuscular Profiles from Force Plate Testing During Elite Women’s Basketball National Team Camps: A Retrospective Comparison by Final Roster Status

**DOI:** 10.3390/sports14020084

**Published:** 2026-02-15

**Authors:** Hiroki Ogata, Kazuya Yamazaki, Tomohiro Usui, Kotaro Shinchi, Katsuya Ikeda, Frederick James Henderson, Daichi Yamashita

**Affiliations:** 1 Graduate School of Physical Education, National Institute of Fitness and Sports in Kanoya, Kagoshima 891-2311, Japan; ogatahiroki@hotmail.co.jp; 2 Japan Basketball Association, Tokyo 112-0004, Japan; 3 Japan Institute of Sports Sciences, Japan High Performance Sport Center, Tokyo 115-0056, Japanwillpower.c@me.com (K.I.); frederick.henderson@jpnsport.go.jp (F.J.H.); 4 Faculty of Sport Science, Nippon Sport Science University, Tokyo 158-8508, Japan

**Keywords:** elite female basketball players, isometric mid-thigh pull, countermovement jump, portable force plate

## Abstract

This study compared force plate-derived lower-limb strength and power metrics between selected and non-selected female basketball players for major international competitions. Thirty-two female players attending the final national team camps for the 2022 World Cup and the 2024 Olympic Games completed isometric mid-thigh pull (IMTP) and countermovement jump (CMJ) testing on dual force plates (1000 Hz). IMTP peak force, rate of force development (RFD) over 0–200 and 0–250 ms, CMJ height, and phase-specific kinetic variables were compared between roster (*n* = 14) and non-roster (*n* = 18) players. Eleven roster players had previous World Cup/Olympic experience (1.5 ± 1.2 selections across all 14 players), whereas non-roster players had none. The roster group was older than the non-roster group (26.8 ± 4.2 vs. 22.3 ± 3.1 years, *p* = 0.002); therefore, between-group comparisons were adjusted for age and playing position using analyses of covariance (ANCOVAs). After adjustment, no between-group differences were observed in IMTP- or CMJ-derived performance outcomes (all *p* ≥ 0.12; partial η^2^ = 0.00–0.09). Therefore, in this elite cohort, roster status did not reflect force plate metrics but may reflect factors beyond these tests, including age and prior international experience.

## 1. Introduction

Basketball is a high-intensity intermittent sport demanding substantial lower-limb force and power production underpinning critical actions such as sprinting, jumping, or change of direction [1,2]. In female cohorts, these demands may be compounded by sex-related differences in biomechanical loading and neuromuscular control that can manifest in test-derived performance characteristics. For example, such differences have been reported in CMJ force–time/phase characteristics (e.g., braking and propulsive phase kinetics) and lower-limb muscle activation patterns during jump, which may influence force expression and jump performance [3,4]. Sex-related differences have also been reported in IMTP peak force and rapid force production characteristics, indicating potential differences in maximal and time-sensitive force expression during isometric tasks [5]. Within the high-stakes environment of national team selection camps, these physiological factors are further challenged as athletes must maintain peak neuromuscular performance despite condensed competitive schedules and significant training load accumulation. Therefore, clarifying the characteristics of lower-limb force production during national team preparations is critical for contextualizing neuromuscular readiness and informing targeted support strategies to ensure optimal roster readiness.

The expected association between competitive level and lower-limb strength may be obscured by the homogeneity of elite national team rosters. To date, however, research directly examining these qualities in elite female basketball players is limited. Previous studies examining differences in competitive level have reported that National Collegiate Athletic Association (NCAA) Division I players outperform National Association of Intercollegiate Athletics (NAIA) players in countermovement jump (CMJ) performance [6], as do Women’s National Basketball Association (WNBA) athletes compared to Women’s National Basketball League (WNBL) athletes [7]. However, these findings may not fully translate to the unique environment of national team selection. While candidates are selected from a restricted pool of world-class players, they arrive from a ‘patchwork’ of professional clubs with disparate training philosophies and cumulative loads. Consequently, the extent to which lower-limb strength characteristics vary within such high-performing cohorts demands clarification.

Addressing this gap requires navigating the unique logistical landscape of elite international preparation, where time and equipment are limited. In high-performance settings, portable force plate assessments using the isometric mid-thigh pull (IMTP) and countermovement jump (CMJ) have emerged as a popular alternative to traditional laboratory testing [8,9,10,11]. While IMTP provides a leg-dominant measure of maximal isometric strength and rate of force development, CMJ captures lower-limb ballistic performance [12,13]. Notably, by documenting biomechanical strategies, eccentric–concentric coupling during a CMJ offers insight into an athlete’s readiness for the demands of elite basketball that jump height alone cannot provide [12,13]. Importantly, portable force plates allow for the rapid evaluation of lower-limb kinetics via a small number of maximal efforts performed directly on-court with high reliability [14,15]. Determining whether these metrics—traditionally used for monitoring readiness—show significant differences based on selection outcomes would provide a data-driven framework for assembling elite female rosters within the condensed timelines of international competition [16].

Understanding that physical capacity may influence roster status while elite athletes represent a physically homogeneous population, the purpose of this study was to investigate whether IMTP- and CMJ-derived metrics differed between players later classified as roster vs. non-roster for major international competitions (FIBA Women’s World Cup and Olympic Games). We hypothesized that players selected for the final roster would display higher IMTP- and CMJ-derived metrics than non-selected players, albeit by a small margin.

## 2. Materials and Methods

### 2.1. Participants

Thirty-two female basketball players (8 world-class and 24 elite/international level [17]) who attended national team training camps in preparation for the 2022 World Cup (*n* = 20 tested) and/or the 2024 Olympic Games (*n* = 20 tested) completed force plate testing as part of the team assessment (age = 24.3 ± 4.2 years, height = 174.6 ± 9.3 cm, body mass = 68.8 ± 8.7 kg). Eight players completed testing in both the 2022 and 2024 camps; for these players, only the 2022 data and corresponding roster classification were used to avoid duplicate observations and maintain statistical independence. Thus, the earliest available observation was retained to minimize potential bias associated with greater accumulated national team experience and selections in later camps. Players were included in the study if they were free from injury at the time of testing, able to perform maximal efforts, and completed both the IMTP and CMJ assessments. The roster group comprised 14 players (11 backcourt and 3 frontcourt), and the non-roster group comprised 18 players (9 backcourt and 9 frontcourt). Eleven roster players had previous World Cup/Olympic experience (1.5 ± 1.2 selections across all 14 players), whereas non-roster players had none.

The study was approved by the Institutional Ethics Committee of the Japan Institute of Sports Sciences (No. 2021-057-3). All tests were conducted at the beginning of a training session during the national team training camps as part of the team assessment. Prior to testing, all participants were informed of the potential benefits and risks of the tests, and written consent was obtained regarding the potential use of their data for research purposes. Information on the study’s purpose and the opt-out option was posted on the institute’s website (https://www.jpnsport.go.jp/hpsc/business/ourwork/tabid/1322/Default.aspx, accessed on 1 December 2025), allowing athletes to opt out without facing any disadvantages.

### 2.2. Setting and Equipment

Testing was conducted in a training facility with a hard rubber floor. Ground reaction forces were collected using dual uniaxial force plates (Hawkin Dynamics Inc., G3 Force Plates, Westbrook, ME, USA) sampling at 1000 Hz. Prior to testing, the system was zeroed and calibrated according to the manufacturer’s instructions and the plates were securely fixed to prevent any movement and to ensure consistent contact with the floor. Data were recorded using the manufacturer’s software (Hawkin Dynamics Cloud, version 10.0.1) and exported as CSV files for subsequent analyses. Outcome variables were derived using the manufacturer’s default processing workflow (see Ref. [18] for methodological details). The system has been shown to provide valid measurements compared to gold-standard methods [9,19], supporting their use in this study.

### 2.3. Isometric Mid-Thigh Pull (IMTP)

The IMTP testing followed the protocol recommended by Comfort et al. [8]. The IMTP was performed on the FPs using a portable isometric pull rack with bar-height adjustment in 5 cm steps (Hawkin Dynamics Inc., Westbrook, ME, USA). Joint angles were visually verified using a custom-made transparent angle template with thick reference lines marking the target knee (125–145°) and hip (140–150°) angle ranges. The trunk was maintained upright, with the scapulae retracted and depressed, the shoulders aligned vertically or slightly posterior to the bar. Feet were positioned approximately hip-width apart, and the bar remained in contact with the mid-thigh. Lifting straps were used during maximal trials to minimize the influence of grip strength.

Following a general warm-up, body weight was first recorded using the system’s “weigh-in” mode while participants stood still on the FPs; the lowest 1 s average of the vertical GRF was used [20]. Participants then completed three familiarization IMTP trials at approximately 50%, 75%, and 90% of perceived maximum effort, each lasting 3 s. For maximal trials, a standardized external focus cue was given: “push the ground as fast and as hard as possible.” Participants assumed the starting position with minimal pre-tension, removed slack, and remained motionless before performing a 3 s maximal pull upon hearing the countdown “3–2–1 GO”. Trials showing countermovement or excessive pre-tension were excluded. IMTP outcomes were peak force and time-interval average RFD over 0–100, 0–150, 0–200, and 0–250 ms (RFD_100_, RFD_150_, RFD_200_, RFD_250_, respectively). At least two valid trials were performed, and their mean values were used for analysis to improve reliability [21].

### 2.4. Countermovement Jump (CMJ)

Participants performed several submaximal CMJs for familiarization. During test trials, they placed their hands on their hips to restrict arm swing and were instructed to “quickly dip down to around 90° of knee flexion and jump as high as possible” [22]. Countermovement depth, particularly knee flexion angle, was visually assessed by the examiner, and trials that deviated from the criteria were repeated. Verbal feedback on jump height was provided to each athlete between attempts. The force plate system was zeroed before each athlete’s first trial. Each athlete performed three trials with approximately 60 s of rest between attempts. The following countermovement jump (CMJ) variables were used for analysis: jump height, jump momentum, countermovement depth, modified reactive strength index (mRSI: calculated as jump height divided by time to takeoff), time to takeoff, takeoff velocity, peak relative braking force, peak relative braking power, braking rate of force development (RFD), peak relative propulsive force, and peak relative propulsive power. At least three valid trials were performed, and their mean values were used for analysis to provide a more reliable and practically relevant performance metric [23].

### 2.5. Statistical Analysis

Statistical analyses were conducted using IBM SPSS Statistics (Version 30.0; IBM Corp., Armonk, NY, USA). Statistical significance was set at *p* < 0.05.

Within-session reliability was evaluated using a two-way random-effects intraclass correlation coefficient with absolute agreement for the average of k trials (ICC_2,k_; k = 3 for CMJ and k = 2 for IMTP). Normality of the pooled data used for the reliability analyses was assessed using the Shapiro–Wilk test and Q–Q plots. Because one variable (jump momentum) deviated from normality (Shapiro–Wilk test, *p* < 0.05) and the sample size was limited, 95% confidence intervals (CIs) for ICCs were obtained using bootstrap resampling (2000 resamples of participants with replacement). ICC point estimates were calculated from the original dataset. Reliability was considered acceptable for ICC_2,k_ > 0.75 [16] and coefficient of variation (CV) < 10%.

Between-group differences in participant characteristics (age, height, and body mass) between roster and non-roster players were examined using independent-samples *t*-tests. Due to the relatively small sample size within groups (*n* < 18), Hedges’ g was used to calculate the measure of effect size (i.e., g = 0.2 is a small, g = 0.5 is a moderate, and g > 0.8 is a large [24]).

Because roster and non-roster players differed in age and showed an imbalanced distribution of playing positions, and because both factors may influence IMTP- and CMJ-derived strength and power outcomes, adjusted between-group comparisons were conducted using separate analysis of covariance (ANCOVAs). Playing position was included as a fixed factor and age as a covariate to adjust for potential confounding. Model assumptions were evaluated using residual diagnostics. Residual normality was evaluated using the Shapiro–Wilk test and Q–Q plots. No marked deviations from normality were detected. Partial eta squared (η^2^) was reported as the effect size for the ANCOVA models, interpreted as small = 0.01–0.06, moderate = 0.06–0.14, or large ≥ 0.14 [24]. Adjusted mean differences, derived from estimated marginal means, and their 95% CIs were obtained using bias-corrected and accelerated (BCa) bootstrap resampling (2000 resamples of participants with replacement).

## 3. Results

Descriptive statistics and within-session reliability for all measurements are presented in Table 1. All variables demonstrated acceptable levels of reliability (ICC_2,k_ = 0.84–1.00 and CV ≤ 8.25%) except RFD_100_ and RFD_150_ in IMTP and braking RFD in CMJ (ICC_2,k_ = 0.64, 0.84, and 0.97; CV = 18.79%, 11.06%, and 12.00%, respectively). Accordingly, RFD100, RFD150, and braking RFD were excluded from subsequent analyses.

Participant characteristics by roster status are shown in Table 2. The roster group was significantly older than the non-roster group (26.8 ± 4.2 vs. 22.3 ± 3.1 years, *p* = 0.002, Hedges’ g = 1.19).

Adjusted group comparisons for IMTP- and CMJ-derived outcomes are summarized in Table 3. Although the ANCOVA analysis showed small-to-moderate differences between group, these differences were not significant (all *p* ≥ 0.12; partial η^2^ = 0.00–0.09). Overall, adjusted mean differences across variables were small and their BCa bootstrap 95% CIs spanned zero (Table 3).

## 4. Discussion

This study compared lower-limb strength and power characteristics between elite female basketball players classified as roster or non-roster for major international tournaments using IMTP and CMJ. Although the roster players were older than the non-roster players, roster status was not associated with any IMTP- or CMJ-derived metric after adjusting for age and playing position. Therefore, within a high-level and otherwise relatively homogeneous cohort, force plate-derived strength and power metrics do not clearly differentiate roster from non-roster status.

All IMTP- and CMJ-derived variables demonstrated adequate reproducibility, except for RFD_100_, RFD_150_ and braking RFD. Because early IMTP RFD and CMJ braking RFD are short-window force–time slope measures, onset/phase-boundary error and noise are amplified, often compromising absolute reliability [25,26]. Thus, they should be used cautiously for monitoring.

In the present study, no statistically detectable differences in IMTP-derived variables were observed between roster and non-roster players. The IMTP peak relative force (roster: 37.7 N/kg; non-roster: 35.2 N/kg) exceeded the value of 27 N/kg reported for NCAA Division I female basketball players by Townsend et al. [27] and was comparable to the 38.3 N/kg reported for NCAA Division I female guards by Uysal et al. [6]. RFD values (RFD_200_, RFD_250_; 89, and 83 N/s/kg, respectively) were also higher than the mean values reported for NCAA Division I female players (approximately 38, and 40 N/s/kg) [27], illustrating the elite athletic level of the present cohort. However, in that previous study, participants performed the IMTP in their preferred second-pull power-clean position by self-selecting their hip and knee angles [27], which may have underestimated peak force and RFD compared with the standardized method used in the present study [8]. Therefore, direct comparisons are challenging, and the higher values observed here may reflect methodological differences rather than necessarily superior strength. Our IMTP results suggest that players invited to the final national team camp possess a homogeneously high level of maximal strength, regardless of selection status. Thus, although IMTP-derived strength indices may be a prerequisite for roster players, they do not serve as decisive discriminators between roster and non-roster status. It should be noted, however, that there are no published data on IMTP performance in women’s national team basketball players from other countries, making it difficult to determine whether the strength levels observed in the present study are sufficient to underpin elite-level performance. Future research involving similarly high-level elite female basketball players is needed to establish benchmark values for lower-limb strength.

As with the IMTP variables, no statistically detectable between-group differences were observed for all CMJ-derived indices between the roster and non-roster groups. This contrasts with a previous study reporting CMJ differences between higher- and lower-minute NCAA Division I players [28]. Kraemer et al. [29] and Philipp et al. [28] reported CMJ values of ~34 cm (jump height), 0.50 (mRSI), and ~52 W/kg (peak relative propulsive power) in NCAA Division I female players, whereas roster players in the present study showed 28.8 cm, 0.34, and 44.1 W/kg, respectively. CMJ height in the Brazilian women’s national team reported by Freire et al. [30] (≈27–28 cm), assessed using a comparable force plate CMJ protocol, was similar to the present value. Nevertheless, these cross-study comparisons should be interpreted cautiously because the present cohort represents a different competitive level and selection context. Also, jump height estimation differed across studies (flight time in Freire et al. vs. impulse–momentum in the present study), which may introduce systematic differences [31]. Accordingly, the comparable or slightly lower CMJ height observed here may reflect differences in training focus, population characteristics, and/or protocol-specific factors across cohorts. Therefore, beyond a minimum threshold of jumping ability, roster outcomes in the national team context may be driven more by basketball-specific factors than by CMJ performance alone.

In terms of mean age, based on the rosters for the women’s basketball tournament at the Paris Olympic Games [32], the present national team (28.8 years) was comparable to the other 11 countries (25.8–30.1 years). In this context, the finding that only roster players in the present study had world-stage experience is consistent with previous reports indicating that coaches tend to rely more on experienced players in major international tournaments [33]. Therefore, the absence of differences in lower-limb strength and power indices between roster and non-roster players in the present study suggests that, at the selection stage, technical, perceptual–cognitive, and tactical factors—such as three-point shooting ability, tactical understanding, and experience in international competition—may have been more important than lower-body physical capacities. In international games, aspects that depend heavily on experience, including tactical comprehension, decision-making under pressure, and leadership, have been suggested to influence game performance. However, these factors were not measured in the present study, and we therefore cannot determine how they contributed to roster decisions. Future studies incorporating such measures are needed to examine how they relate to roster outcomes.

Among the limitations of this study, first, let us mention the modest sample size, which limits statistical power to detect group differences. Second, this retrospective, cross-sectional comparison does not allow evaluation of within-athlete changes over time or training responses (i.e., longitudinal adaptations), which may be particularly relevant in national team environments. Third, data were collected across different camp years (2022 and 2024), and year-to-year variation in training status, acute fatigue, and testing context may have influenced outcomes. Although models adjusted for age and playing position, residual confounding remains possible. Fourth, acute fatigue status and prior training/match load were not standardized or fully quantified at the time of testing; thus, differences (or the absence of differences) may partly reflect transient readiness rather than stable neuromuscular characteristics. Nonetheless, the present data, collected in the immediate pre-competition period for major international tournaments within an extremely high-quality and homogeneous group of players, provide valuable insights into the lower-limb strength and power characteristics of female basketball athletes on the international stage.

At the national team level, force plate metrics from IMTP and CMJ may be interpreted as prerequisite or monitoring tools. Practitioners (e.g., strength and conditioning coach) can use these tests to confirm that athletes meet a high neuromuscular capacity baseline during congested camps, detect meaningful deviations from an athlete’s typical profile, and individualize strength–power maintenance strategies, while recognizing that selection results likely reflect an integration of experience and basketball-specific competencies alongside physical capacities.

## 5. Conclusions

We examined lower-limb strength and power characteristics in elite female basketball players using IMTP- and CMJ-derived metrics, comparing players classified as roster versus non-roster. Adjusted ANCOVA models accounting for age and playing position showed no roster-status differences for any force plate-derived outcome. These findings indicate that force plate-derived lower-limb strength and power metrics did not clearly differentiate roster from non-roster players, suggesting that differences in roster status may be more closely related to basketball-specific factors (e.g., technical and tactical qualities) and competitive experience than to force plate metrics. However, given that age confounded roster status in this cohort, these results require cautious interpretation. Future research integrating longitudinal designs and technical–tactical metrics is warranted to more fully explain elite selection.

## Figures and Tables

**Table 1:** Descriptive statistics and within-session reliability for isometric mid-thigh pull (IMTP) and countermovement jump (CMJ) variables.

Variables	Mean ± SD	ICC_2,k_ (95% CI)	CV%
IMTP			
Peak force (N/kg)	36.39 ± 4.41	0.97 (0.93, 0.99)	2.45
RFD_100_ (N/s/kg)	89.91 ± 22.42	0.64 (0.39, 0.79)	18.79
RFD_150_ (N/s/kg)	91.50 ± 20.22	0.84 (0.69, 0.92)	11.06
RFD_200_ (N/s/kg)	90.84 ± 18.03	0.91 (0.83, 0.96)	6.78
RFD_250_ (N/s/kg)	81.66 ± 15.90	0.95 (0.90, 0.98)	4.96
CMJ			
Jump height (m)	0.274 ± 0.041	0.99 (0.97, 0.99)	2.74
Jump momentum (kg∙m/s)	158.59 ± 19.78	0.99 (0.99, 1.00)	1.37
Countermovement depth (m)	−0.331 ± 0.041	0.94 (0.88, 0.97)	4.49
mRSI	0.34 ± 0.07	0.97 (0.94, 0.98)	5.95
Time to takeoff (s)	0.82 ± 0.10	0.92 (0.86, 0.95)	5.01
Takeoff velocity (m/s)	2.31 ± 0.17	0.99 (0.98, 0.99)	1.37
Peak braking force (N/kg)	21.78 ± 2.51	0.95 (0.91, 0.97)	3.80
Peak braking power (W/kg)	−19.28 ± 3.70	0.92 (0.84, 0.95)	8.25
Braking RFD (N/s/kg)	72.65 ± 31.38	0.97 (0.93, 0.98)	12.00
Peak propulsive force (N/kg)	22.36 ± 1.87	0.95 (0.90, 0.97)	2.74
Peak propulsive power (W/kg)	42.36 ± 5.23	0.99 (0.97, 0.99)	1.86

Values are presented as mean ± SD. IMTP, isometric mid-thigh pull; CMJ, countermovement jump; RFD, rate of force development; mRSI, modified reactive strength index; ICC, intraclass correlation coefficient; CI, confidence interval; CV, coefficient of variation.

**Table 2:** Participant characteristics by final roster status.

Variable	Roster (*n* = 14)	Non-Roster (*n* = 18)	*p*	Hedges’ g (95% CI)
Age (years)	26.8 ± 4.2	22.3 ± 3.1	<0.01	1.19 (0.44, 1.93)
Height (cm)	173.0 ± 9.8	175.9 ± 8.9	0.39	−0.30 (−0.99, 0.38)
Body mass (kg)	67.7 ± 10.5	69.7 ± 7.2	0.53	−0.22 (−0.90, 0.47)

Values are presented as mean ± SD.

**Table 3:** Adjusted comparisons of IMTP- and CMJ-derived outcomes by final roster status (roster−non-roster).

				ANCOVA		
Variable	Roster (*n* = 14)	Non-Roster (*n* = 18)	Adjusted Mean Difference (Bootstrap BCa 95% CI)	F	*p*	Partial η^2^
IMTP						
Peak force (N/kg)	37.90 ± 4.10	35.22 ± 4.46	1.39 (−2.19, 4.64)	0.64	0.43	0.02
RFD_200_ (N/s/kg)	89.50 ± 16.07	91.89 ± 19.82	−3.95 (−20.10, 10.40)	1.46	0.24	0.05
RFD_250_ (N/s/kg)	83.45 ± 14.37	80.27 ± 17.27	−0.47 (−13.86, 11.46)	2.00	0.17	0.07
CMJ						
Jump height (m)	0.288 ± 0.041	0.264 ± 0.040	0.027 (−0.031, 0.081)	0.21	0.65	0.01
Jump momentum (kg∙m/s)	160.16 ± 26.87	157.37 ± 12.56	21.96 (−2.04, 43.26)	0.34	0.57	0.01
Takeoff velocity (m/s)	2.37 ± 0.16	2.27 ± 0.17	0.11 (−0.12, 0.32)	0.20	0.66	0.01
Time to takeoff (s)	0.86 ± 0.10	0.80 ± 0.10	0.06 (−0.06, 0.22)	0.47	0.50	0.02
mRSI	0.34 ± 0.07	0.34 ± 0.08	0.01 (−0.08, 0.08)	0.51	0.48	0.02
Countermovement depth (m)	−0.341 ± 0.032	−0.323 ± 0.051	−0.03 (−0.06, 0.00)	0.18	0.67	0.01
Peak braking force (N/kg)	21.08 ± 2.68	22.33 ± 2.29	−1.12 (−3.69, 1.04)	2.52	0.12	0.09
Peak braking power (W/kg)	−17.79 ± 3.60	−20.45 ± 3.43	1.17 (−2.30, 5.47)	0.91	0.35	0.03
Peak propulsive force (N/kg)	22.08 ± 1.73	22.58 ± 1.99	−0.64 (−2.67, 1.06)	1.51	0.23	0.05
Peak propulsive power (W/kg)	44.06 ± 5.13	41.04 ± 5.04	3.15 (−2.85, 8.61)	0.08	0.78	0.00

IMTP, isometric mid-thigh pull; CMJ, countermovement jump; RFD, rate of force development; mRSI, modified reactive strength index.

## Data Availability

The data presented in this study are available on request from the corresponding author; however, the data are not publicly available due to privacy and ethical restrictions related to elite athlete monitoring.

## Abbreviations

The following abbreviations are used in this manuscript:

CMJ	countermovement jump
COD	change of direction
IMTP	isometric mid-thigh pull
mRSI	modified reactive strength index
RFD	rate of force development
ANCOVA	analysis of covariance
